# Perceived Social Support, Shame, and Psychopathological Symptoms After Perinatal Loss in Portuguese Women

**DOI:** 10.3390/ejihpe16010010

**Published:** 2026-01-05

**Authors:** Mariana Ribeiro, Paula Saraiva Carvalho, Ana Torres, Dário Ferreira

**Affiliations:** 1 Department of Psychology and Education, University of Beira Interior, 6200-209 Covilhã, Portugal; psc@ubi.pt (P.S.C.); ana.carla.torres@ubi.pt (A.T.); 2 Research Center in Sports Sciences, Health Sciences and Human Development (CIDESD), University of Beira Interior, 6200-209 Covilhã, Portugal; 3 RISE-Health, Department of Medical Sciences, Faculty of Health Sciences, University of Beira Interior, 6200-209 Covilhã, Portugal; 4 Department of Mathematics and Center of Mathematics, University of Beira Interior, 6200-209 Covilhã, Portugal; dario@ubi.pt

**Keywords:** perceived social support, shame, psychopathological symptoms, perinatal loss

## Abstract

(1) Background: Perinatal loss is a deeply painful and often invisible experience, with a significant impact on mental health. This study aimed to assess levels of psychopathological symptoms, shame, and perceived social support according to the type of perinatal loss; explore the relationships between these variables; and analyze the mediating effect of perceived social support on the relationship between shame and symptoms, as well as the moderating effect of the type of loss. (2) Methods: A total of 501 Portuguese women who had experienced perinatal loss participated in the study, recruited through an online questionnaire. Psychopathological symptoms, shame, perceived social support, and type of loss were assessed. Analyses included descriptive statistics, Spearman correlations, normality and homogeneity of variances tests, and mediation and moderation models with PROCESS. (3) Results: The results revealed high levels of anxiety and depression, and moderate levels of shame. Perceived social support, especially from partners and family members, was high. Shame correlated positively with symptoms and negatively with social support. Only social support from friends significantly mediated the relationship between shame and psychological distress. (4) Conclusions: These results reinforce the protective role of support networks and the importance of clinical interventions focused on reducing shame.

## 1. Introduction

Perinatal loss is a tragically common experience with a significant emotional impact and is often socially devalued. Although there is no consensus in the literature on its definition, we consider perinatal loss to include situations of spontaneous abortion (early and late), fetal death and neonatal death (early and late), as well as medical and voluntary terminations of pregnancy (Public Health Agency of Canada, 2020). The literature shows that one in four pregnancies ends before 28 weeks of gestation, with 2.6 million fetal deaths worldwide (World Health Organization, 2024). In Portugal, there were 473 fetal and neonatal deaths in 2024 (PORDATA, 2024), with no data available on the prevalence of spontaneous abortions in the country.

In addition to suffering the loss of their baby, women often experience a lack of recognition of their grief, which exacerbates the psychological vulnerability associated with this type of event (Lobb et al., 2010). The grief experienced after perinatal loss is often neglected or invalidated and not legitimised by health professionals, family members or society in general (Duncan & Cacciatore, 2015). Those experiencing this type of grief often report feelings of social isolation, guilt, personal responsibility and shame (Burden et al., 2016). Following a pregnancy loss, women often report feelings of failure stemming from the perception that their bodies were unable to produce a healthy baby (Frøen et al., 2011). Additionally, many express doubts about their ability to sustain a subsequent pregnancy and deliver a live, healthy child (Cacciatore, 2009). While this emotional experience is often interpreted as guilt, feelings of failure are more consistent with theoretical definitions of shame (Gilbert, 2002; Goss & Allan, 2009). Within the evolutionary framework proposed by Gilbert (2002), shame is conceptualised as a ‘self-conscious emotion’, as it is associated with how the individual believes they are perceived by others. It involves feelings of inadequacy, undesirability, defect, worthlessness and powerlessness (Gilbert, 2002). Previous studies have identified shame as a relevant predictor of psychopathology, significantly impacting depression and anxiety (Blackmore et al., 2011; Kim et al., 2011).

Research has shown that perinatal loss is often associated with high levels of psychopathological symptoms, such as depression, anxiety and somatic symptoms. These symptoms can persist for months or even years after the loss (Irmscher et al., 2024; Jacob et al., 2017; Lewkowitz et al., 2019). Conversely, social support is widely recognised as a key protective factor for mental health following loss (Cacciatore et al., 2009). Social support is generally conceptualised as a set of interpersonal interactions and resources offering emotional, instrumental or informational assistance to individuals experiencing adversity (Iwanowicz-Palus et al., 2021b). It encompasses dimensions of social integration, reciprocity, and trust in meaningful relationships, enabling individuals to feel understood, valued, and supported (Iwanowicz-Palus et al., 2021a). Perceiving emotional support from family, friends or professionals can mitigate psychological distress and promote adaptation (Kay et al., 2024; Razurel et al., 2017). However, not all sources of support are perceived as effective, and many women report feeling misunderstood or isolated, particularly when the loss occurs in the early stages of pregnancy (Monteiro, 2020).

Despite their relevance, few studies have explored the relationships between shame, perception of social support and psychopathological symptoms following perinatal loss, particularly in Portugal. Furthermore, the type of perinatal loss experienced (miscarriage, fetal death or neonatal death) may affect the emotional response and perception of support. The present study therefore aims to assess levels of psychopathological symptoms, shame, and perceived social support, depending on the type of perinatal loss experienced; analyze the relationships between these variables; and to explore the mediating effect of perceived social support on the relationship between shame and psychopathological symptoms, as well as the moderating effect of the type of perinatal loss on this association.

## 2. Materials and Methods

Regarding sociodemographic data, the age of Portuguese participants (*n* = 501) ranged from 21 to 47 years (M = 34.00, SD = 4.77; *n* = 501). Adult women who had experienced perinatal loss were eligible to complete the questionnaire if they met the following inclusion criteria: (1) having suffered a perinatal loss, (2) being 18 years of age or older, (3) being a woman and (4) being of Portuguese nationality. Participants were not eligible if they (1) have not experienced a perinatal loss; (2) are of a nationality other than Portuguese, (3) male and (4) aged above 49 years (decline in fertility) (Kocourková et al., 2015; PORDATA, 2023).

Most participants were married (49.9%, *n* = 250) or living in a civil partnership (42.1%, *n* = 211), had attended higher education (75.4%), were employed (95.6%), had an average socioeconomic status (57.3%), and lived in a large city (41.5%). Regarding clinical characteristics, the results showed that most women reported having had a perinatal loss prior to participating in this study (67.1%, *n* = 336), the majority of which were early miscarriages (63.1%, *n* = 316). Most of the perinatal losses in the sample occurred in 2023 (46.3%, *n* = 232), with 29.7% reporting that they did not know the cause of their loss. 34.5% (*n* = 173) of participants reported receiving some type of support (psychological or psychiatric) at the time of responding to the questionnaire. The final sample size (*n* = 501) exceeded the recommended minimum for this type of analysis, as sample sizes between 150 and 400 are considered acceptable by authors such as Schoemann et al. (2017).

This study was approved by the Ethics Committee of the University of Beira Interior (CE-UBI-Pj-2022-066). Data collection took place between June 2023 and June 2024, using an online questionnaire developed on the Microsoft Forms platform. The access link was shared through social networks, associations, and projects dedicated to supporting mothers and fathers bereaved by perinatal loss, as well as in person at the Cova da Beira Hospital in Covilhã. The questionnaire included an introduction with information about the research and its objectives, followed by questions about sociodemographic characteristics and clinical aspects related to pregnancy and the experience of perinatal loss. To ensure eligibility, the questionnaire included specific questions about the experience of perinatal loss, such as the type of loss and the year it occurred. Only participants who provided clear, consistent information about a perinatal loss were included in the analysis. All responses were manually reviewed by members of the research team, and cases with incomplete or inconsistent information were excluded. The Microsoft Forms platform was configured to allow only one response per user, and the date and time of submission were automatically recorded.

Specific scales were also applied to assess the variables under analysis. Participation was entirely voluntary and anonymous, with all participants having previously accepted informed consent. Recruitment through both hospitals and perinatal loss associations, as well as online dissemination, allowed access to women at different stages and types of perinatal loss, ensuring sample heterogeneity while preserving anonymity in a sensitive research context.

The Portuguese version of the 18-item Psychopathological Symptom Inventory (BSI-18) was used to assess recent psychopathological symptoms. This instrument identifies signs of psychological distress in three distinct domains: depression, anxiety and somatization. Participants indicated the intensity with which they had experienced each symptom over the previous seven days using a response scale ranging from 0 (not at all) to 4 (extremely) (Canavarro et al., 2017). In this study, the inventory demonstrated satisfactory internal consistency, with a Cronbach’s alpha of α = 0.93. The Multidimensional Scale of Perceived Social Support (MSPSS) was used to evaluate participants’ perceptions of social support from three main sources: family, friends, and significant others. This scale contains 12 items organised into three subscales of four items each and a total social support measure. Responses are given on a 7-point Likert scale ranging from 1 (strongly disagree) to 7 (strongly agree). In this study, the reliability coefficients (Cronbach’s α) obtained were as follows: α = 0.94 for the Friends subscale, α = 0.96 for the Family subscale, α = 0.95 for Significant Others and α = 0.95 for the total scale. The Shame and Guilt Scale of the Personal Attitudes Questionnaire was originally developed by Harder and Greenwald (1999) and later adapted for the Portuguese population by Geada (2003). This scale consists of 22 items, which are assessed using a 5-point Likert scale ranging from 0 (never feel) to 4 (always or almost always feel). For this study, only the shame subscale was used, which showed an internal consistency of α = 0.87.

Statistical analysis was performed using Statistical Package for the Social Sciences (SPSS) software, version 29.1. In this study, descriptive analyses were used to characterize the sample and the variables under study. Normality and homogeneity were assessed using the Kolmogorov–Smirnov and Levene tests, whose results indicated an absence of normal distribution and a lack of homogeneity in several variables, so non-parametric methods were chosen. Spearman’s correlation was used to analyze the associations between psychopathological symptoms, shame, and perceived social support. Finally, mediation and moderation analyses were conducted using regression models, allowing us to verify the mediating role of perceived social support in the relationship between shame and psychopathological symptoms, considering the type of perinatal loss as a moderating variable.

## 3. Results

### 3.1. Sources of Perceived Social Support

**Figure 1:**
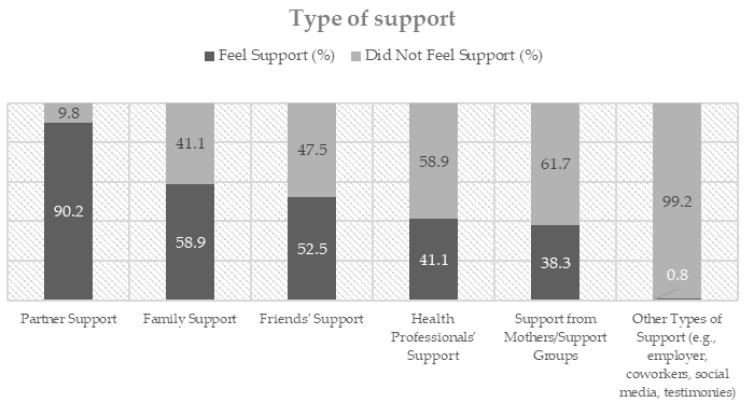
Perceived types of social support after perinatal loss.

The perception of support received from different sources following perinatal loss is illustrated in Figure 1. The data reveal that partners were the greatest source of support, with 90.2% of participants reporting that they felt supported by their partner. Family support also stood out as relatively strong, with 58.9% of participants indicating that they felt supported. Friends (52.5%) and healthcare professionals (41.1%) were also identified as important sources of support. By contrast, only 0.8% reported receiving support from other sources (e.g., employers, co-workers, social networks, and testimonies from other mothers), while support from mothers and support groups was reported by even fewer participants (38.3%).

### 3.2. Descriptive Statistics

Descriptive statistics for the study variables are summarised in Table 1. The results indicated that the sample exhibited psychopathological symptoms above the expected average for a non-clinical population, particularly among women who had experienced a late miscarriage (M = 21.53, SD = 1.41). The mean shame score for this group was 14.01 (SD = 0.81), suggesting moderate levels of shame. Across all subscales of perceived social support, participants reported moderate to high mean scores, varying according to the type of loss experienced. The overall score also reflected a generally high level of perceived social support among participants.

### 3.3. Correlations

As shown in Table 2, all variables exhibited statistically significant correlations. Shame exhibited a weak and negative association with perceived social support, particularly with respect to the family dimension (r = −0.274, *p* < 0.001), as well as with friends (r = −0.296, *p* < 0.001) and significant others (r = −0.293, *p* < 0.001). The strongest correlation was observed with the total MSPSS scale (r = −0.324, *p* < 0.001). Conversely, shame revealed a positive and moderate association with psychopathological symptoms, particularly anxiety (r = 0.443, *p* < 0.001), depression (r = 0.436, *p* < 0.001), somatization (r = 0.386, *p* < 0.001), and notably with the BSI-18 Overall Severity Index (r = 0.476, *p* < 0.001). The correlations between perceived social support and psychopathological symptoms were all negative and statistically significant. Perceived social support from family members showed a weak correlation with anxiety (r_s_ = −0.254, *p* < 0.001) and somatization (r_s_ = −0.170, *p* < 0.001), and a moderate correlation with depression (r_s_ = −0.368, *p* < 0.001). Similarly, perceived support from friends showed a weak negative correlation with somatization (r = −0.190, *p* < 0.001) and a moderate correlation with anxiety (r = −0.315, *p* < 0.001) and depression (r = −0.347, *p* < 0.001). The ‘significant others’ dimension followed an identical pattern. The total perceived social support scale correlated negatively with all psychopathological symptom dimensions, with the strongest association being with depression (r = −0.403, *p* < 0.001), followed by the global symptom index (r = −0.359, *p* < 0.001), anxiety (r = −0.314, *p* < 0.001) and somatization (r = −0.205, *p* < 0.001). These results suggest that higher levels of perceived social support are associated with fewer psychological symptoms, whereas higher levels of shame are associated with greater emotional distress and lower levels of perceived social support.

### 3.4. Mediation and Moderation Analyses

**Figure 2:**
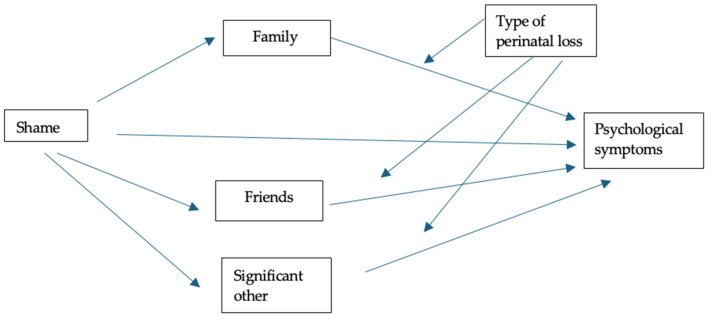
Testing the mediating effect of perceived social support on the relationship between feelings of shame and psychopathological symptoms, with the type of perinatal loss as a moderator.

Figure 2 presents the results of a mediation analysis which tested the role of perceived social support in the relationship between shame and psychopathological symptoms, considering the type of perinatal loss as a moderating variable. The results showed that the perception of social support from friends only presented a significant indirect effect (β = 0.0469, SE = 0.0235; 95% CI [0.0062; 0.0984]). However, support from family (β = 0.0379, SE = 0.0242; 95% CI [−0.0062; 0.0896]) and significant others (β = 0.0187, SE = 0.0312; 95% CI [−0.0422; 0.0817]) did not have a statistically significant indirect effect. Before the introduction of the mediating variables, it is important to note that the total effect of shame on psychopathological symptoms was significant (β = 0.9646, SE = 0.0706; 95% CI [0.8252; 1.1039]). Following the introduction of the mediators, the direct effect remained statistically significant (β = 0.8612, SE = 0.0719; 95% CI [0.7199; 1.0025]). These results confirm that shame partially mediates the relationship between social support and psychopathological symptoms; greater social support is associated with lower levels of shame and consequently lower symptomatology. Furthermore, social support was found to have a direct effect in reducing psychological distress, suggesting that it acts both directly and indirectly. However, the type of perinatal loss did not play a significant moderating role, meaning that the strength of these relationships remains relatively stable regardless of whether the loss occurred in the early or late stages of pregnancy or in the neonatal period.

## 4. Discussion

This study aimed to assess levels of psychopathological symptoms, shame and perceived social support depending on the type of perinatal loss experienced. It also aimed to analyze the relationships between these variables and explore the mediating effect of perceived social support on the relationship between shame and psychopathological symptoms. The study examined a sample of Portuguese women who had experienced perinatal loss. The results showed that the participants exhibited psychopathological symptoms that exceeded the expected average for a non-clinical population, particularly within the group that had experienced late miscarriage. This group also exhibited moderate levels of shame. Higher levels of perceived social support were also found to be associated with fewer symptoms, while higher levels of shame were associated with greater emotional distress. Support from friends was the only significant mediator of the relationship between shame and symptoms, suggesting a specific protective role for this source of support. These results further our understanding of the emotional and relational factors involved in perinatal grief, emphasizing the importance of intervention strategies that promote diverse, sensitive support networks for these women.

### 4.1. Psychopathological Symptoms

In our study, we found clinically significant levels of psychopathological symptoms, particularly among women who had experienced a late miscarriage, fetal death or neonatal death. A study by Bennett et al. (2008) corroborates these findings, revealing that women who had experienced perinatal loss exhibited higher-than-expected levels of anxiety and depression compared to a non-clinical normative sample (Derogatis, 1993). The gestational age at which loss occurs may therefore determine the intensity and duration of the psychological impact experienced by women. Empirical evidence shows that early pregnancy losses, particularly in the first trimester, are often associated with high levels of anxiety and depressive symptoms in the weeks immediately following the loss (Tsartsara & Johnson, 2006). However, in cases of early pregnancy loss, depressive symptoms tend to decrease progressively over time, with many women recovering within a year (Gísladóttir, 2016). However, our study revealed that women who experienced later perinatal losses, such as late miscarriage, stillbirth and neonatal death, demonstrated more intense and prolonged grief reactions accompanied by more severe psychopathological symptoms, including anxiety, depression and complicated grief (Clauss, 2009; Mendes et al., 2023). This disparity can be explained by several psychological factors, such as greater emotional investment in pregnancy, a more advanced gestational stage, and available coping mechanisms. The emotional bond with the baby intensifies as pregnancy progresses, making the grieving process more complex and worsening symptoms of psychological distress (Díaz-Pérez et al., 2023). Another factor that exacerbates grief in late-term losses is the attribution of blame and shame, both of which were found to be prevalent in the late-term miscarriage group in the present study. Women who attribute the loss to their own behaviours or personal characteristics tend to experience higher levels of psychological distress, exacerbating symptoms of anxiety and depression (K. R. Barr et al., 2024; Clauss, 2009). By contrast, women who experience early-term miscarriages, despite also suffering significant emotional distress, tend to engage in lower levels of self-blame, which can mitigate the severity of the psychological impact (Lok & Neugebauer, 2007).

### 4.2. Shame

Another finding of our study was the presence of shame, specifically at moderate levels, in the group that experienced late miscarriage. In the context of loss, this emotion may arise from the perception of personal failure, often manifesting as feelings of bodily inadequacy or an inability to fulfil societal and cultural expectations of motherhood (P. Barr & Cacciatore, 2008). Furthermore, shame demonstrated positive and moderate correlations with all dimensions of psychopathological symptomatology, reinforcing its relevance as an emotional risk factor, as evidenced by the existing scientific literature (Kim et al., 2011). In their meta-analysis review, Kim et al. (2011) demonstrated that shame was strongly associated with depressive symptoms. According to Kim et al. (2011), shame may be more strongly related to depressive symptoms in women than in men. This is because women tend to invest more in interpersonal relationships and construct their identity based on social and relational roles, such as motherhood (Kim et al., 2011). Given that shame is often related to threats to social acceptance and belonging, it is plausible that it plays a more central role in the presence of depressive symptoms among women. Pineles et al. (2006) found that individuals who are more susceptible to feelings of shame tend to report higher levels of anxiety and somatization.

Additionally, the study found a negative correlation between shame and perceived social support. This corroborates the existing literature which states that the stigma associated with perinatal loss can trigger feelings of shame, leading to bereaved parents becoming isolated from their social support networks (Bhat & Byatt, 2016). However, the study by Caldwell et al. (2023) also demonstrated that low levels of social support can intensify feelings of shame. Another factor, such as the emotional security experienced in interpersonal relationships, may account for these results. Studies by Akbag and Imamoglu (2010) and Wei et al. (2005) suggest that individuals with insecure relationships tend to report higher levels of shame. ‘For most women, at a time when they desperately need love and support, the taboo, secrecy and shame surrounding miscarriage can make them feel extremely lonely’ (Feasey, 2019).

### 4.3. Perceived Social Support

When asked who had provided them with the most support after experiencing a perinatal loss, participants generally reported high levels of support from various sources, with support from partners standing out as the main source, followed by support from family and friends. This finding is consistent with the existing literature, which identifies the partner (typically male) as the most significant source of support for bereaved women (Meaney et al., 2017; Wagner et al., 2018). During perinatal loss, women often turn to their partners for support to cope with emotional distress, particularly when they feel that other members of their support network do not understand or show interest (Corbet-Owen, 2003). By sharing the experience of loss, partners play an essential role in providing emotional support and helping to cope with grief, thereby maintaining the stability of the relationship (Corbet-Owen, 2003). According to research (Corbet-Owen, 2003; Sutan & Miskam, 2012), women who have experienced perinatal loss seek support from family and friends to feel accepted and respected. This provides these mothers with emotional security and understanding.

The negative correlation between perceived social support and psychopathological symptoms suggests a protective effect, particularly about depressive symptoms. This is supported by the research of Razurel et al. (2017) and Kay et al. (2024), which revealed that perceiving social support can reduce depressive symptoms. Perceiving higher levels of social support has been found to be associated with lower levels of psychopathological symptoms as it acts as a protective factor against stressful situations (Thomas et al., 2022). This support provides emotional and practical resources that enhance coping mechanisms and resilience, thereby alleviating stress and grief symptoms (Sarper & Rodrigues, 2024).

The mediation analysis revealed that social support from friends was the only factor that significantly mediated the relationship between shame and psychopathological symptoms. In situations of perinatal loss, family and partners may constitute sources of social pressure or minimization of pain for some women, which can compromise their perception of support in these contexts (Bennett et al., 2005; Cacciatore, 2013). Furthermore, previous studies have shown that social support is not homogeneous, with different sources having different effects on psychological adjustment (Thoits, 2011). Regarding perinatal loss specifically, research indicates that support from friends is frequently linked to increased emotional validation and reduced depressive and anxious symptoms (Cacciatore et al., 2009). However, the existing literature suggests that family support can be ambivalent, oscillating between caring attitudes and invalidating, judgmental responses from bereaved families (Jones et al., 2019). Moreover, the effectiveness of social support depends on its availability, perceived quality, and suitability to the individual’s emotional needs (Iwanowicz-Palus et al., 2021a). Specifically, regarding their partner, men tend to exhibit different forms of emotional expression after experiencing perinatal loss (Obst et al., 2020). We believe this may partly explain our results. In this sense, although family members and significant others represent a relevant social network, they may not have enough influence to mediate the impact of shame on psychopathological symptoms. The fact that the type of perinatal loss did not moderate the relationship between shame, social support and symptoms suggests that support from friends has a consistent protective effect in different loss contexts, ranging from early miscarriages to neonatal deaths. Previous studies have confirmed that supportive friendship networks reduce depressive and anxious symptoms, regardless of gestational age. This highlights the importance of interventions that promote this type of support (Cacciatore et al., 2009; Razurel et al., 2017).

### 4.4. Limitations and Future Research

This study has some methodological limitations. The sample was collected through online platforms for convenience, so it may not be representative of the general population. Self-reported data are also subject to response bias. Although several procedures were implemented to verify eligibility and data consistency, the use of online self-report measures entails an inherent risk of response bias that should be considered when interpreting the findings. The cross-sectional design of the study prevents causal inferences from being made. Despite these limitations, however, the results offer valuable insights into clinical practice and research in perinatal mental health. Identifying shame as an emotion associated with psychological distress following perinatal loss emphasizes the importance of psychotherapeutic interventions that encourage self-acceptance and challenge internalized beliefs of failure. Evidence that support from friends plays a mediating role between shame and psychological distress reinforces the need for strategies that promote informal support networks. Future research should include longitudinal studies to monitor symptom evolution over time and clarify the observed relationships. Qualitative methodologies should also be integrated to further explore perceptions of the quality of social support and underlying relational dynamics. Finally, we recommend developing and evaluating intervention programmes combining shame-reduction strategies, such as compassion- and acceptance-based approaches, with strengthening informal social networks. These programmes should investigate their impact on the mental health of women bereaved by perinatal loss.

## 5. Conclusions

In this study, we examined the relationships between shame, perceived social support, and psychopathological symptoms in Portuguese women who experienced perinatal loss. The results highlighted that higher levels of shame were associated with greater psychological distress, while perceived social support, particularly from friends, played a protective and mediating role. Although the type of loss did not significantly influence these relationships, support networks emerged as a key factor in coping with grief. These findings emphasize the importance of interventions that reduce shame and strengthen informal social support, contributing to better mental health outcomes for women following perinatal loss. Future research should explore longitudinal designs and qualitative approaches to deepen the understanding of social support dynamics and the development of targeted clinical programs.

## Figures and Tables

**Table 1:** Descriptive statistics: alphas, means, standard deviations of the BSI-18 and its subscales, the Multidimensional Scale of Perceived Social Support and its subscales, and the QAP—Shame Scale.

Type of Perinatal Loss						
	Early Miscarriage (*n* = 316)		Late Miscarriage (*n* = 80)		Fetal and Neonatal Death (*n* = 105)	
Measures	Range (Min–Max)	M(SD)	Range (Min–Max)	M(SD)	Range (Min–Max)	M(SD)
BSI-18						
Anxiety	0.00–23	7.14 (0.27)	0.00–24	8.56 (0.51)	0.00–22	8.23 (0.47)
Depression	0.00–23	7.10 (0.29)	0.00–22	8.42 (0.63)	0.00–20	8.25 (0.47)
Somatization	0.00–17	3.73 (0.21)	0.00–18	4.54 (0.44)	0.00–19	4.15 (0.41)
Global Severity Index	0.00–56	17.98 (0.69)	0.00–56	21.53 (1.41)	0.00–56	20.63 (1.17)
MSPSS						
Family	0.00–7	7.14 (0.27)	0.00–7	5.13 (0.22)	0.50–7	5.41 (0.16)
Friends	0.00–7	7.14 (0.27)	0.00–7	4.71 (0.25)	0.75–7	5.53 (0.14)
Significant Others	0.00–7	7.14 (0.27)	0.00–7	5.93 (0.20)	1.50–7	6.28 (0.11)
Total Scale	0.00–7	7.14 (0.27)	0.00–7	5.26 (0.20)	1.33–7	5.74 (0.12)
QAP—Shame						
Total Scale	0.00–33	7.14 (0.27)	1.00–33	14.01 (0.81)	0.00–33	11.44 (0.60)

**Table 2:** Spearman correlation between psychopathological symptoms (BSI-18), shame (QAP-Shame), and perceived social support (MSPSS).

Variables	1	2	3	4	5	6	7	8	9
1. BSI-18—Anxiety	1								
2. BSI-18—Depression	0.753 **	1							
3. BSI-18—Somatization	0.689 **	0.556 **	1						
4. Global Severity Index (BSI-18)	0.929 **	0.900 **	0.798 **	1					
5. MSPSS—Family	−0.254 **	−0.368 **	−0.170 **	−0.309 **	1				
6. MSPSS—Friends	−0.315 **	−0.347 **	−0.190 **	−0.332 **	0.635 **	1			
7. MSPSS—Significant Others	−0.255 **	−0.325 **	−0.171 **	−0.292 **	0.585 **	0.617 **	1		
8. MSPSS—Total Scale	−0.314 **	−0.403 **	−0.205 **	−0.359 **	0.886 **	0.881 **	0.747 **	1	
9. QAP—Shame	0.443 **	0.436 **	0.386 **	0.476 **	−0.274 **	−0.296 **	−0.293 **	−0.324 **	1

** *p* < 0.001.

## Data Availability

The data supporting the findings of this study are not publicly available due to privacy and ethical restrictions involving sensitive personal information. Data may be available from the corresponding author upon reasonable request and subject to ethical approval.

## References

[B1-ejihpe-16-00010] Akbag M., Imamoglu S. E. (2010). The prediction of gender and attachment styles on shame, guilt, and loneliness. Educational Sciences: Theory & Practice.

[B2-ejihpe-16-00010] Barr K. R., Nguyen T. A., Pickup W., Cibralic S., Mendoza Diaz A., Barnett B., Eapen V. (2024). Perinatal continuity of care for mothers with depressive symptoms: Perspectives of mothers and clinicians. Frontiers in Psychiatry.

[B3-ejihpe-16-00010] Barr P., Cacciatore J. (2008). Problematic emotions and maternal grief. OMEGA-Journal of Death and Dying.

[B4-ejihpe-16-00010] Bennett S. M., Litz B. T., Lee B. S., Maguen S. (2005). The scope and impact of perinatal loss: Current status and future directions. Professional Psychology: Research and Practice.

[B5-ejihpe-16-00010] Bennett S. M., Litz B. T., Maguen S., Ehrenreich J. T. (2008). An exploratory study of the psychological impact and clinical care of perinatal loss. Journal of Loss and Trauma.

[B6-ejihpe-16-00010] Bhat A., Byatt N. (2016). Infertility and perinatal loss: When the bough breaks. Current Psychiatry Reports.

[B7-ejihpe-16-00010] Blackmore E., Côté-Arsenault D., Tang W., Glover V., Evans J., Golding J., O’Connor T. (2011). Previous prenatal loss as a predictor of perinatal depression and anxiety. British Journal of Psychiatry.

[B8-ejihpe-16-00010] Burden C., Bradley S., Storey C., Ellis A., Heazell A. E., Downe S., Cacciatore J., Siassakos D. (2016). From grief, guilt, pain and stigma to hope and pride: A systematic review and meta-analysis of mixed-method research of the psychosocial impact of stillbirth. BMC Pregnancy and Childbirth.

[B9-ejihpe-16-00010] Cacciatore J. (2009). The silent birth: A feminist perspective. Social Work.

[B10-ejihpe-16-00010] Cacciatore J. (2013). Psychological effects of stillbirth. Seminars in Fetal and Neonatal Medicine.

[B11-ejihpe-16-00010] Cacciatore J., Schnebly S., Froen J. F. (2009). The effects of social support on maternal anxiety and depression after stillbirth. Health & Social Care in the Community.

[B12-ejihpe-16-00010] Caldwell J. M., Meredith P., Whittingham K., Ziviani J., Wilson T. (2023). Women pregnant after previous perinatal loss: Relationships between adult attachment, shame, and prenatal psychological outcomes. Journal of Reproductive and Infant Psychology.

[B13-ejihpe-16-00010] Canavarro M. C., Nazaré B., Pereira M., Gonçalves M. M., Simões M. R., Almeida L. (2017). Inventário de sintomas psicopatológicos 18 (BSI-18). Psicologia clínica e da saúde: Instrumentos de avaliação.

[B14-ejihpe-16-00010] Clauss D. K. (2009). Psychological distress following miscarriage and stillbirth: An examination of grief, depression and anxiety in relation to gestational length, women’s attributions, perception of care and provision of information. Doctoral dissertation.

[B15-ejihpe-16-00010] Corbet-Owen C. (2003). Women’s perceptions of partner support in the context of pregnancy loss(es). South African Journal of Psychology.

[B16-ejihpe-16-00010] Derogatis L. R. (1993). BSI brief symptom inventory: Administration, scoring, and procedures manual.

[B17-ejihpe-16-00010] Díaz-Pérez E., Haro G., Echeverria I. (2023). Psychopathology present in women after miscarriage or perinatal loss: A systematic review. Psychiatry International.

[B18-ejihpe-16-00010] Duncan C., Cacciatore J. (2015). A systematic review of the peer-reviewed literature on self-blame, guilt, and shame. OMEGA—Journal of Death and Dying.

[B19-ejihpe-16-00010] Feasey R. (2019). Pregnancy loss: Shame and silence over a shared experience. Infertility and non-traditional family building.

[B20-ejihpe-16-00010] Frøen J. F., Cacciatore J., McClure E. M., Kuti O., Jokhio A. K., Islam M., Shiffman J. (2011). Stillbirths: Why they matter. The Lancet.

[B21-ejihpe-16-00010] Geada M. (2003). Questionário de sentimentos pessoais.

[B22-ejihpe-16-00010] Gilbert P., Gilbert P., Miles J. (2002). Body shame: A biopsychosocial conceptualisation and overview, with treatment implications. Body shame: Conceptualisation, research and treatment.

[B23-ejihpe-16-00010] Gísladóttir S. G. (2016). Prior pregnancy loss: Mental health during and after subsequent pregnancies. Master’s thesis.

[B24-ejihpe-16-00010] Goss K., Allan S. (2009). Shame, pride, and eating disorders. Clinical Psychology & Psychotherapy.

[B25-ejihpe-16-00010] Harder D., Greenwald D. F. (1999). Further validation of the shame and guilt scales of Harder’s Personal Feelings Questionnaire–2. Psychological Reports.

[B26-ejihpe-16-00010] Irmscher L., Marx R., Linke M., Zimmermann A., Drössler S., Berth H. (2024). Anxiety, depression, somatization and psychological distress before and 2–6 years after a late termination of pregnancy due to fetal anomalies. BMC Women’s Health.

[B27-ejihpe-16-00010] Iwanowicz-Palus G., Mróz M., Bień A. (2021a). Quality of life, social support and self-efficacy in women after a miscarriage. Health and Quality of Life Outcomes.

[B28-ejihpe-16-00010] Iwanowicz-Palus G., Mróz M., Bień A., Jurek K. (2021b). Social support and subjective assessment of psychophysical condition, health, and satisfaction with quality of life among women after pregnancy loss. BMC Pregnancy and Childbirth.

[B29-ejihpe-16-00010] Jacob L., Polly I., Kalder M., Kostev K. (2017). Prevalence of depression, anxiety, and adjustment disorders in women with spontaneous abortion in Germany: A retrospective cohort study. Psychiatry Research.

[B30-ejihpe-16-00010] Jones K., Robb M., Murphy S., Davies A. (2019). New understandings of fathers’ experiences of grief and loss following stillbirth and neonatal death: A scoping review. Midwifery.

[B31-ejihpe-16-00010] Kay T. L., Moulson M. C., Vigod S. N., Schoueri-Mychasiw N., Singla D. R. (2024). The role of social support in perinatal mental health and psychosocial stimulation. Yale Journal of Biology and Medicine.

[B32-ejihpe-16-00010] Kim S., Thibodeau R., Jorgensen R. S. (2011). Shame, guilt, and depressive symptoms: A meta-analytic review. Psychological Bulletin.

[B33-ejihpe-16-00010] Kocourková J., Konečná H., Burcin B., Kučera T. (2015). How old is too old? A contribution to the discussion on age limits for assisted reproduction technique access. Reproductive BioMedicine Online.

[B34-ejihpe-16-00010] Lewkowitz A. K., Rosenbloom J. I., Keller M., López J. D., Macones G. A., Olsen M. A., Cahill A. G. (2019). Association between stillbirth ≥ 23 weeks’ gestation and acute psychiatric illness within 1 year of delivery. American Journal of Obstetrics and Gynecology.

[B35-ejihpe-16-00010] Lobb E. A., Kristjanson L. J., Aoun S. M., Monterosso L., Halkett G. K. B., Davies A. (2010). Predictors of complicated grief: A systematic review of empirical studies. Death Studies.

[B36-ejihpe-16-00010] Lok I. H., Neugebauer R. (2007). Psychological morbidity following miscarriage. Best Practice & Research Clinical Obstetrics & Gynaecology.

[B37-ejihpe-16-00010] Meaney S., Corcoran P., Spillane N. (2017). Experience of miscarriage: An interpretative phenomenological analysis. BMJ Open.

[B38-ejihpe-16-00010] Mendes D. C. G., Fonseca A., Cameirão M. S. (2023). The psychological impact of early pregnancy loss in Portugal: Incidence and the effect on psychological morbidity. Frontiers in Public Health.

[B39-ejihpe-16-00010] Monteiro M. S. R. M. (2020). A influência da satisfação conjugal, do apoio social e dos estados emocionais negativos no processo de luto decorrente de uma perda gestacional. Master’s thesis.

[B40-ejihpe-16-00010] Obst K. L., Due C., Oxlad M., Middleton P. (2020). Men’s grief following pregnancy loss and neonatal loss: A systematic review and emerging theoretical model. BMC Pregnancy and Childbirth.

[B41-ejihpe-16-00010] Pineles S. L., Street A. E., Koenen K. C. (2006). The differential relationships of shame-proneness and guilt-proneness to psychological and somatization symptoms. Journal of Social and Clinical Psychology.

[B42-ejihpe-16-00010] PORDATA (2023). Fertility rate by age group.

[B43-ejihpe-16-00010] PORDATA (2024). Óbitos fetais e neonatais.

[B44-ejihpe-16-00010] Public Health Agency of Canada (2020). Family-centred maternity and newborn care: National guidelines *(Chapter 7)*.

[B45-ejihpe-16-00010] Razurel C., Kaiser B., Antonietti J.-P., Epiney M., Sellenet C. (2017). Relationship between perceived perinatal stress and depressive symptoms, anxiety, and parental self-efficacy in primiparous mothers and the role of social support. Women & Health.

[B46-ejihpe-16-00010] Sarper E., Rodrigues D. L. (2024). The role of perceived social support in the grief experiences of more anxious and self-compassionate people. OMEGA—Journal of Death and Dying.

[B47-ejihpe-16-00010] Schoemann A. M., Boulton A. J., Short S. D. (2017). Determining power and sample size for simple and complex mediation models. Social Psychological and Personality Science.

[B48-ejihpe-16-00010] Sutan R., Miskam H. M. (2012). Psychosocial impact of perinatal loss among Muslim women. BMC Women’s Health.

[B49-ejihpe-16-00010] Thoits P. A. (2011). Mechanisms linking social ties and support to physical and mental health. Journal of Health and Social Behavior.

[B50-ejihpe-16-00010] Thomas S., Kanske P., Schäfer J., Hummel K. V., Trautmann S. (2022). Examining bidirectional associations between perceived social support and psychological symptoms in the context of stressful event exposure: A prospective, longitudinal study. BMC Psychiatry.

[B51-ejihpe-16-00010] Tsartsara E., Johnson M. P. (2006). The impact of miscarriage on women’s pregnancy-specific anxiety and feelings of prenatal maternal–fetal attachment during the course of a subsequent pregnancy. Journal of Psychosomatic Obstetrics & Gynaecology.

[B52-ejihpe-16-00010] Wagner N. J., Vaughn C. T., Tuazon V. (2018). Fathers’ lived experiences of miscarriage. The Family Journal.

[B53-ejihpe-16-00010] Wei M., Mallinckrodt B., Larson L. M., Zakalik R. A. (2005). Adult attachment, depressive symptoms, and validation from self versus others. Journal of Counseling Psychology.

[B54-ejihpe-16-00010] World Health Organization (2024). The unacceptable stigma and shame women face after baby loss must end.

